# Predicting Physical Violence Against Corrections Officers Across Three Levels of Severity Using Individual and Environmental Characteristics

**DOI:** 10.1177/08862605241287802

**Published:** 2024-10-11

**Authors:** Samantha S. Taaka, Armon Tamatea, Devon L. L. Polaschek

**Affiliations:** 1 The University of Waikato, Hamilton, New Zealand

**Keywords:** violent offenders, violence exposure, workplace violence

## Abstract

Working in prisons can be a challenging job, managing a population of incarcerated people while keeping oneself, one’s colleagues, and the people themselves safe. Some corrections officers may expect violence in the workplace, yet being a victim of violence is no trivial experience. In prison, violent incidents are categorized according to the severity of the violence perpetrated. However, we do not know how characteristics of a violent incident may contribute to the severity of violence perpetrated toward corrections staff. To begin to address this gap, we examined characteristics of physical assault incidents in New Zealand prisons between 2016 and 2020, in which the perpetrator of the incident was a male prisoner and the victim was a corrections officer. We examined the prediction of incidents across three levels of severity using individual and environmental characteristics. Perpetrators of serious violence tended to be already segregated from the general population at the time of the assault. We also found that perpetrators of assault against staff were different from the general prison population: prisoners who assaulted staff were more likely to be younger, gang affiliated, and had higher security classifications compared to prisoners who did not assault staff. Research suggests that characteristics of perpetrators can contribute to their risk of perpetrating violence; we found that characteristics of perpetrators (i.e., being segregated) can also contribute to the severity of violence perpetrated. Furthermore, we offer a direct comparison between prisoners who assaulted staff and prisoners who did not, therefore cementing research that prisoners who assaulted staff are different from the rest of the prison population.

Most people do not go to work expecting to be verbally and physically assaulted. For corrections officers who work in prisons, verbal abuse, threats, and risk of physical harm are facets of everyday work experience. Corrections officers undertake one of the most dangerous occupations; they need to be hypervigilant in order to manage the safety of incarcerated people, while also maintaining their own safety and that of colleagues (Ellison & Caudill, 2020; Steiner & Woolredge, 2020). The occurrence of prisoner assaults on staff is related to the characteristics of the perpetrators, the victims, and the environments in which violence occurs (McNeeley, 2021; Sorensen et al., 2011; Steiner & Woolredge, 2020). However, there is limited research about whether these characteristics differ depending on how *seriously* a prisoner assaults staff. In this study, we use a centralized database of physical assault incidents toward staff in New Zealand prisons to examine perpetrator and environmental characteristics of assault incidents at three levels of incident severity.

Many corrections officers have roles that require them to continuously interact with prisoners, enforce rules, and mediate interactions between prisoners when there is potential or actual conflict (Ellison & Caudill, 2020). As a result, corrections officers are employed in one of the most dangerous occupations (Gordon et al., 2012); they experience high rates of physical injuries and violent victimization in the workplace across an international scope (Duhart, 2001; Giannini, 2021; Gordon et al., 2012; Ministry of Justice, 2023). In New Zealand, physical violence against corrections officers has been increasing (0.18 serious assaults on staff per 100 prisoners in 2018, compared to 0.30 serious assaults on staff per 100 prisoners in 2023; Ara Poutama Aotearoa, 2022; Cook, 2020) and corrections officers’ levels of stress are also increasing, partly because of their concerns about being victims of violence (Christian, 2018). Some corrections officers have described the tense environment within prisons as a “pressure cooker” and reported fearing for their safety (Christian, 2018).

Corrections officers report high rates of Post-traumatic stress disorder (PTSD), and high levels of: stress, burnout, symptoms of depression and anxiety, stress-related health problems, and negative coping strategies (e.g., substance abuse; Boudoukha, et al., 2012; Fusco et al., 2012; Jaegers et al., 2020, 2022; Kinman et al., 2017; Lambert et al., 2010; Spinaris et al., 2012; Steiner & Woolredge, 2015). In addition to the negative consequences outlined above, corrections officers also have limited options for avoiding activities or places where they may no longer feel safe, like victims of violence in the general community may (Spinaris et al., 2012). Corrections officer roles often require contact with prisoners, which may be distressing for staff who have been assaulted by a prisoner (Ellison & Caudill, 2020; Spinaris et al., 2012), and may cause them to feel fearful or unsafe at work (Gordon et al., 2012). To manage feeling unsafe, staff may avoid work or resign from their position, contributing to understaffing (Lambert et al., 2010).

## Predictors of Prisoner–Staff Assaults

Prisons can be viewed as ecosystems, where characteristics across multiple levels interact in the occurrence of an incident of physical assault (Tamatea et al., 2023). Characteristics at the individual level (i.e., individual characteristics of the perpetrator and victim) interact with characteristics at the relationship level (i.e., the relationship between perpetrator and victim), which are encompassed in characteristics of the spatial and temporal environment of the prison. Various variables from different parts of the ecosystem have been examined previously (Lahm, 2009; Steier & Woolredge, 2020); we briefly review a selection of perpetrator, victim, and environmental characteristics that are relevant to this study.

## Perpetrator Characteristics

Common characteristics of prisoners who are likely to assault staff include: being younger, having a minority ethnic identity, and having a history of violent behavior (Arbach-Lucioni et al., 2012; McNeeley, 2021; Sorensen et al., 2011). Gang membership also increases a prisoner’s likelihood of committing violence in prison. On average, gang members have higher levels of violent misconduct, including physical assault against other prisoners and staff members, compared to people who are not gang members (Brabyn et al., 2023; Pyrooz et al., 2011; Sorensen et al., 2024; Worrall & Morris, 2012).

## Victim Characteristics

Prison violence can occur after considerable planning or may unfold rapidly and spontaneously, and perpetrators of violence may rationally decide who to assault based on the characteristics of their potential victims (Sorensen et al., 2011; Steiner & Woolredge, 2020). Corrections officers are more likely to be assaulted by a prisoner if they are men, younger, and have less experience in their role (Sorensen et al., 2011; Steiner & Woolredge, 2017). Younger and less experienced staff may be perceived as more vulnerable due to being less confident in their role compared to their older, more experienced counterparts (Steiner & Woolredge, 2017). Men are more likely than women to be assaulted by prisoners (Cashmore et al., 2012; Konda et al., 2013; Sorensen et al., 2011; Wolff et al., 2007) because women may more commonly than men use verbal strategies to de-escalate situations before the progress to physical violence (Dennehy & Nantel, 2006). On the other hand, men may be more likely to be assaulted by prisoners who adhere to traditional gender roles (Crewe, 2006). In a traditional gender-role perspective, men may disapprove of physical violence toward women (Crewe, 2006), and therefore, male staff may be considered a more acceptable target of violence.

## Population-Based, Environmental, and Situational Characteristics

Several population-level characteristics may be related to risk of staff assaults, although relevant research mainly focuses on prison violence in general, rather than specifically toward staff. The security classification for a unit or prison is one such population feature. Because security classification is based on risk factors for violence such as age, offense history, and risk of escape, higher security prisons contain prisoners with higher levels of estimated violence risk (Ara Poutama Aotearoa, n.d.). Because these prisoners have a higher estimated risk of perpetrating violence, staff who are tasked with managing them are already at higher risk of violence victimization. But the regimes themselves may also contribute to violence.

The rate of prisoner turnover is an example of a regime-based factor; the stability of the residents in a prison relative to the volume of new prisoners entering and leaving may also contribute to the general level of tension and potential for violence in a prison (Baggio et al., 2018, 2020). Higher rates of turnover in a prison or unit are associated with higher levels of violent infractions, prisoner self-harm and prisoner suicide (Baggio et al., 2018, 2020; van Ginneken et al., 2017).

Other characteristics of the regime can also contribute toward physical violence. Times of day when prisoners are “unlocked” and activities when prisoners have more opportunity to move around the prison and interact with corrections officers (e.g., recreation, employment) may be riskier. For example, in some prisons, riskier times include the afternoons when prisoners may have more free time (McNeeley, 2021) or alternatively when prisoner’s cells are first unlocked in the morning (Sorensen et al., 2011).

## Severity of Prisoner–Staff Assaults

Although considerable research has been conducted on prison violence, including violence toward staff, the relative severity of incidents has not been subject to much research attention. One study (Cunningham & Sorensen, 2007) suggested that some characteristics of perpetrators (i.e., age and criminal history) are associated with more serious incidents of general prison violence. They examined violence perpetrated by close custody prisoners (i.e., prisoners maintained within an armed perimeter or under direct armed supervision when outside of a secure perimeter). They found that prisoners who were younger, had previously perpetrated violence in prison, and who were convicted of a violent crime were significantly more likely to commit assaults resulting in serious injuries compared to older prisoners, prisoners who had not previously perpetrated violence in prison, and who were not convicted of a violent crime. However, there is limited research about whether characteristics of perpetrators are associated with more serious outcomes specifically in relation to assaults on staff, nor has previous research compared predictors of different levels of prisoner assault severity.

## Current Study

This study uses official incident data recorded by corrections officers, to examine the environmental, situational, and perpetrator variables related to assaults on corrections officers perpetrated by a single male prisoner. Our aims were to (a) describe the characteristics of assault incidents and their perpetrators, (b) examine their similarities and differences across three levels of assault severity, and (c) compare the characteristics of assault perpetrators with the remainder of the prison population.

## Method

### Data Source

Ara Poutama Aotearoa (the New Zealand Department of Corrections) operates 18 adult prisons for 8,118 prisoners (as of June 2023). Sentenced prisoners are managed across five security levels (Minimum, Low, Low-Medium, High, Maximum^1^), but 43.4% of prisoners are on remand awaiting conviction and sentencing. They are managed in high-security units. Corrections officers routinely document incidents concerning prisoner behavior (Ara Poutama Aotearoa, n.d.). Incident reports are legal documents and form a record of prisoners’ experiences and behavior during their time in custody. Incidents range from the mundane (e.g., routinely escorting a prisoner to the dentist) to serious events (e.g., medical emergencies, accidents, and assaults). We examined all incidents comprising a physical assault of a corrections officer by a prisoner that corrections officers recorded between 2016 and 2020 in the centralized electronic database,

The initial dataset consisted of 3,392 such incidents. We removed those incidents in which the role of the prisoner was unknown or listed as the victim of the incident (*n* = 112); those in which the perpetrator was a woman (*n* = 365); incidents with multiple perpetrators (*n* = 1,212); incidents of a sexual nature (*n* = 39); and incidents with insufficient information about the perpetrator, such as their security classification or age (*n* = 17), leaving a final dataset of 1,647 incidents in which a single male prisoner physically assaulted a corrections officer within a New Zealand prison. We excluded incidents in which the perpetrator was a woman, or there were multiple perpetrators, or the incident was sexual in nature because the complexity of these characteristics warrant more consideration and analysis than we could cover in the scope of this paper. Men make up 94% of New Zealand’s prisoner population, and most incidents were perpetrated by a lone individual, so we selected our dataset with the intention of understanding physical incidents perpetrated by a male prisoner acting alone. No details were available about the corrections officers who were the victims of the incidents.^2^

Three types of physical assault incidents were recorded: *No Injury, Non-Serious*, and *Serious* incidents. Staff allocate a severity level to the incident based on the severity of the officer’s injuries and the degree of medical attention required to treat the injuries. In No Injury incidents, the victim sustained no injuries as a result of the physical assault. In Non-Serious incidents, the victim’s injuries were treated with basic first aid (e.g., superficial cuts, bruises). In Serious incidents, injuries included fractures, loss of consciousness, and concussions, and required off-site outpatient care, or hospitalization (Ara Poutama Aotearoa, 2022).

The database included perpetrator characteristics (e.g., age, ethnicity, security classification, adjudication status, gang affiliation, RoC*RoI score [see below]). Security classification refers to the current level of supervision deemed appropriate by Ara Poutama Aotearoa to contain a sentenced prisoner, based on the internal and external risk posed (minimum, low, low-medium, high, and maximum; Ara Poutama Aotearoa, n.d.). The adjudication status of the perpetrator referred to whether the perpetrator was serving his sentence, or was still on remand awaiting trial or sentencing at the time of the incident (i.e., remand-accused, or remand-convicted). The gang affiliation of a prisoner was recorded by staff if the prisoner disclosed gang connections, their behavior indicated gang connections (e.g., frequent associations with known gang members in the prison exercise yard), or their person or property was associated with gang connections (e.g., tattoos, clothing, other personal property; Ara Poutama Aotearoa, n.d.). Staff may also identify prisoners as gang affiliated if information is received from communications disclosures (e.g., Intel, mail, and visitors). The RoC*RoI is an actuarial risk assessment tool based on social and demographic variables (i.e., gender, age, age at first offence, and length of time between offenses) used by Ara Poutama Aotearoa that estimates the likelihood of an individual returning to prison in the next 5 years for a new conviction (low, medium, or high risk; Bakker et al., 1999; Johnston, 2021).

Environmental variables included incident information (e.g., time of day, location of the incident). The location of the incident was coded as private (i.e., in or directly around cells) or common areas (i.e., the receiving office, visits). Incidents that had no location information were coded as missing. For incidents that occurred in private locations, the database contained information about the security classification of the unit in which the incident occurred. We were also able to access additional officially recorded information about the unit the perpetrator resided in at the time of the assault, including an estimate of the turnover in the unit. Turnover was measured for each unit in which the incident’s perpetrator resided at the time of the incident, and was the rate at which the population of the unit renewed; a higher rate of turnover indicated less stability in the population of the unit.^3^ We were also able to access an average rate of turnover across all units in New Zealand, between 2016 and 2020. All variables based on information not directly related to the incident itself were documented monthly and were extracted from the nearest record prior to the assault (i.e., not more than 30 days before the incident).

Because prison populations are highly variable between years or even months, we calculated an average number of people in prison at any one time (which we have called “Total Population” for simplicity) to compare to our sample of prisoners who assaulted corrections officers. To make this comparison, we first calculated the population characteristics for each month in the sample time frame (60 months total, between 2016 and 2020); then, we averaged each variable across the 60 months. For example, we calculated the proportion of prisoners who were European for each month between 2016 and 2020; then, we calculated the average of those proportions. The total population comparison group included all men who had been housed in a New Zealand prison between 2016 and 2020 for any duration of remand or sentence (*n* = 9,202), who did not physically assault a staff member and were therefore not in the staff-assault perpetrator sample.

### Analytic Plan

We used IBM SPSS Statistics version 28 (IBM Corp. 2021) for all analysis. First, we conducted descriptive analyses on the complete cleaned incident dataset (*n* = 1,647) to identify the common characteristics of prisoner–staff physical assault incidents. Second, we conducted bivariate analyses to examine associations between person and incident characteristics, and the severity of incidents. To address our third aim, we compared the prisoners who assaulted corrections officers with the remaining total population of the prison during that period who had no recorded incident of corrections officer assault.

## Results

### Characteristics of Perpetrators Who Physically Assaulted Staff

The 1,647 physical incidents were perpetrated by 1,087 men who were housed in a New Zealand prison for some period of time between January 2016 and December 2020. Almost three quarters of perpetrators committed a single assault, almost a quarter of perpetrators committed fewer than five assaults, and 3% of perpetrators committed five assaults or more. Table 1 depicts the characteristics of the assault perpetrators, first by the total sample, then by the severity of the incident. As shown in the first column of Table 1, a majority of the sample were Māori, and approximately one-fifth were European.

**Table 1.** Perpetrator and Environmental Characteristics, by Total Incident Sample and Incident Severity Subsamples.

Variable	Total		No Injury (*n* = 1,009)		Non-Serious (*n* = 584)		Serious (*n* = 54)		F	*df*	*p*
	*M*	*SD*	*M*	*SD*	*M*	*SD*	*M*	*SD*			
Age of perpetrator	29.56	9.22	29.79	9.47	29.21	8.85	29.00	8.46	.846		.429
Perpetrator RoC*RoI	0.64	0.20	0.65	0.19	0.64	0.21	0.63	0.24	.326		.722
	*n*	%	*N*	%	*n*	%	*n*	*%*	*χ* ^2^	*df*	*p*
Ethnicity									19.06	8	.015
Māori	985	59.8	604	59.9	347	59.4	34	63.0			
European	337	20.5	208	20.6	124	21.2	5	9.3			
Pasifika	266	16.2	171	16.9	84	14.4	11	20.4			
Other	38	2.3	17	1.7	17	2.9	4	7.4			
Not recorded	21	1.3	9	0.9	12	2.1	0	0.0			
Gang affiliated	829	50.3	498	49.4	297	50.9	34	63.0	3.99		.143
Adjudication status									33.93	4	<.001
Sentenced	848	51.5	573	56.8	248	42.5	27	50.0			
Remand—accused	576	35.0	325	32.2	234	40.1	17	31.5			
Remand—convicted	223	13.5	111	11.0	102	17.5	10	18.5			
Security classification of prisoner									56.77	10	<.001
Minimum	28	1.7	21	2.1	7	1.2	0	0.0			
Low	56	3.4	37	3.7	16	2.7	3	5.6			
Low medium	166	10.1	121	12.0	39	6.7	6	11.1			
High	390	23.7	282	27.9	96	16.4	12	22.2			
Maximum	167	10.2	93	9.2	68	11.6	6	11.1			
Unclassified	840	51.0	455	45.1	358	61.3	27	50.0			
Voluntary segregation	371	22.5	232	23.0	132	22.6	7	13.0	2.96	2	.228
Directed segregation	630	38.3	336	33.3	257	44.0	37	68.5	39.61	2	<.001
Time of day									13.46	10	.199
00:00–03:59	21	1.3	16	1.6	4	0.7	1	1.9			
04:00–07:59	53	3.2	31	3.1	21	3.6	1	1.9			
08:00–11:59	727	44.1	428	42.4	269	46.1	30	55.6			
12:00–15:59	520	31.6	319	31.6	185	31.7	16	29.6			
16:00–19:59	298	18.1	201	19.9	92	15.8	5	9.3			
20:00–23:59	28	1.7	14	1.4	13	2.2	1	1.9			
Location									10.74	4	.030
Private	1,411	85.7	878	87.0	486	83.2	47	87.0			
Common areas	116	7.0	57	5.6	57	9.8	2	3.7			
Missing	120	7.3	74	7.3	41	7.0	5	9.3			
Characteristics of private incidents	*M*	*SD*	*M*	*SD*	*M*	*SD*	*M*	*SD*	*F*		*p*
Turnover of unit	0.38	0.19	0.36	0.18	0.41	0.18	0.28	0.22	27.44	2	<.001
	*n*	%	*N*	%	*n*	%	*n*	%			
Security classification of remand units									3.99	6	.678
Low medium	12	1.5	4	0.9	8	2.4	0	0.0			
High	438	54.8	239	54.8	183	54.5	16	59.3			
Maximum	29	3.6	18	4.1	10	3.0	1	3.7			
Missing	320	40.1	175	40.1	135	40.2	10	37.0			
Security classification of sentenced units									27.05	8	<.001
Minimum	3	0.4	2	0.3	1	0.4	0	0.0			
Low medium	138	16.3	104	18.2	24	9.7	10	37.0			
High	345	40.7	245	42.8	92	37.1	8	29.6			
Maximum	142	16.7	91	15.9	47	19.0	4	14.8			
Missing	220	25.9	131	22.9	84	33.9	5	18.5			

*Note*. Remand prisoners do not have an allocated security classification. They are usually housed separately from sentenced prisoners. *SD* = standard deviation.

We did not have unit information about incidents that occurred in common areas, including the turnover and security classification of the unit. Some of these incidents occurred in spaces shared by multiple units, or where prisoners from multiple units might be mixing (e.g., kitchens, program areas).

The security classification of the units in which the incident occurred may differ from the security classification of the perpetrator: prisoners may be placed in units with a higher classification than their own due to availability of beds in other units.

As shown in the first column of Table 1, the mean age of perpetrators was 29.56 years (range 17–81). Half of perpetrators were recorded as gang affiliated. The majority were sentenced, with just under half on custodial remand (i.e., pre-conviction or convicted and awaiting sentencing) at the time of the assault. More than half of the men did not have a security classification, mostly because only sentenced prisoners are assigned a security level. Of the men with a security classification, most were classed as high security. More than a fifth of men were in voluntary segregation—where prisoners are segregated from the main prisoner population at their own request, but can circulate freely with other segregated prisoners—and over a third were in directed segregation (DS)—where the prisoner is segregated by prison management, in response to concerns about the risk a prisoner may pose to others—at the time of the assault.

### Characteristics of Incidents

Almost two-thirds of incidents were classified as No Injury (n = 1,009; 61.3%), followed by Non-Serious (n = 584; 35.5%). Serious incidents accounted for fewer than 4% of the total incidents (n = 53; 3.3%). The majority of incidents occurred in private locations within the prison (i.e., in or directly around cells). The remaining incidents occurred in common areas (e.g., in the Receiving Office where prisoners are received into the prison on arrival, the visits room) or the location information was missing. For all incidents that occurred in a private location, more than half occurred in high-security units for Remand prisoners and two-fifths occurred in high-security units for Sentenced prisoners.^4^ The most common time of day for incidents to occur was between 8 am and 12 pm, followed by between 12 pm and 4 pm.

### Perpetrator, Unit, and Incident Characteristics by Severity of Incidents

As shown in Table 1, there were significant relationships between the severity of the incident and the following perpetrator characteristics: perpetrator ethnicity, adjudication status, and whether the perpetrator was in DS. Compared to the overall sample and to perpetrators of less serious incidents, Pasifika prisoners and prisoners who fit in the Other ethnicity category were overrepresented as perpetrators of Serious incidents.^5^ Sentenced prisoners were overrepresented as perpetrators of No Injury incidents compared to prisoners on Remand. Staff also appeared to be at significantly increased risk of serious assault from DS prisoners; more than two thirds of the perpetrators of Serious incidents were already in DS—which is typically used for prisoners where there is a concern that they are at risk from or pose a risk to other prisoners—prior to the incident. In comparison, one-third of perpetrators of No Injury incidents and fewer than half of perpetrators of Non-Serious incidents were in DS prior to the incident occurring.

We also found significant relationships between the severity of the incident and the following environmental variables: the location of the incident, security classification of units that house sentenced perpetrators, and the turnover rate of the unit in which the perpetrator lived. Serious incidents were less likely to occur in common areas compared to No Injury and Non-serious incidents. No Injury and Non-serious incidents were most likely to occur in high-security units, and Serious incidents were most likely to occur in Low Medium units. There was no relationship between the unit security classification of units that housed remand prisoners and incident severity. The units in which Non-Serious incidents occurred had the highest average rate of prisoner turnover, followed by units where No Injury incidents occurred; units in which Serious incidents occurred had the lowest average rate of turnover.

### Comparisons Between Men Who Assaulted Staff and the Total Population

Table 2 depicts the averaged characteristics of the total population of prisoners with no staff assault incident record in comparison to the perpetrators of staff assault. There was a significant difference between the two groups in their age, ethnicity, gang affiliation proportion, adjudication status, and security classification. Notably, one quarter of the total population were gang-affiliated, compared to half of the staff-assault perpetrators. The total population tended to have lower security classifications than staff assault perpetrators at the time of their assault, and the total population had a higher proportion of sentenced prisoners. The total population was also older than staff-assault perpetrators; prisoners in the 20- to 24-year-old age band were particularly overrepresented as staff assault perpetrators compared to their proportion in the total population.

**Table 2.** Comparisons Between Perpetrators of Staff Assault and the Total Prison Population.

Variable	Staff Assault Perpetrators (*n* = 1,647)		Total Population (*n* = 9,202)		*t*	*df*	*p*
	*M*	*SD*	*M*	*SD*			
Age	29.56	9.22	37.15	12.45	23.61	10,847	<.001
Roc*RoI	0.64	0.20	0.47	0.22	29.27	10,847	<.001
	*n*	%	*n*	%	χ2		*p*
Ethnicity of perpetrator					140.48	4	<.001
Māori	985	59.8	4,684	50.90			
European	337	20.5	3,006	32.67			
Pasifika	266	16.2	1,041	11.32			
Other	38	2.3	471	5.12			
Not recorded	21	1.3	0	0.0			
Gang affiliated	829	50.3	2,303	25.02	436.93	1	<.001
Adjudication status					202.51	2	<.001
Sentenced	848	51.5	6,321	68.69			
Remand—accused	576	35.0	1,876	20.39			
Remand—convicted	223	13.5	1,005	10.92			
Security classification					1833.62	5	<.001
Minimum	28	1.7	2,034	22.10			
Low	56	3.4	1,499	16.28			
Low medium	166	10.1	1,729	18.79			
High	390	23.7	914	10.23			
Maximum	167	10.2	0	0.0			
Remand	840	51.0	2,905	31.57			
Age of perpetrator					373.21	6	<.001
<20	120	7.3	220	2.39			
20–24	458	27.8	1,104	12.00			
25–29	384	23.3	1,655	17.99			
30–39	467	28.4	2,803	30.46			
40–49	145	8.8	1,892	20.56			
50–59	61	3.7	989	10.74			
60+	12	0.7	537	5.83			

*Note*. SD = standard deviation.

We conducted a binary logistic regression to examine which prisoner characteristics, if any, were predictive of a prisoner perpetrating a physical assault against a corrections officer (shown in Table 3). We found that the overall model was significant, and that age category, the security classification of the prisoner, and whether the prisoner was gang affiliated significantly predicted perpetration of violence against a corrections officer. Prisoners older than 40 years old (compared to prisoners aged 20 or younger) and lower security prisoners (i.e., prisoners who were minimum, low or low medium security, compared to prisoners who were high security) had significantly lower odds of perpetrating physical assault against a corrections officer. Prisoners who were gang affiliated had significantly higher odds of perpetrating physical assault against a corrections officer, compared to prisoners who were not gang affiliated. Odds ratios are reported in full in Table 3.

**Table 3.** Binary Logistic Regression Using Prisoner Characteristics to Predict Perpetration of Physical Assault Against Corrections Officers.

Prisoner characteristics	*B*	SE	Wald	*p*	OR [95% CI]
Age category					
Under 20			31.28	<.001	
20–24	−0.72	0.85	0.72	.40	0.49 [0.09, 2.57]
25–29	−1.22	0.83	2.17	.14	0.29 [0.06, 1.5]
30–39	−1.45	0.82	3.12	.08	0.23 [0.05, 1.17]
40–49	−2.33	0.84	7.58	.01	0.1 [0.02, 0.51]
50–59	−2.78	0.88	9.94	<.001	0.06 [0.01, 0.35]
60 and over	−3.21	1.05	9.29	<.001	0.04 [0.01, 0.32]
Security classification					
High			80.49	<.001	
Low	−2.55	0.46	30.66	<.001	0.08 [0.03, 0.19]
Low medium	−1.42	0.42	11.55	<.001	0.24 [0.11, 0.55]
Minimum	−4.25	0.50	71.65	<.001	0.01 [0.01, 0.04]
Unclassified	−0.57	0.81	0.49	.48	0.56 [0.11, 2.78]
Ethnicity					
European			1.34	.72	
Māori	0.31	0.29	1.13	.29	1.36 [0.77, 2.38]
Other	−0.10	0.61	0.03	.87	0.91 [0.27, 3.02]
Pacific peoples	0.13	0.42	0.10	.75	1.14 [0.5, 2.6]
Custody status					
Sentenced			0.72	.70	
Remand accused	0.38	0.78	0.23	.63	1.46 [0.32, 6.67]
Remand convicted	0.06	0.82	0.00	.94	1.06 [0.21, 5.32]
Gang affiliation	0.92	0.33	8.03	<.001	2.52 [1.33, 4.77]
Model	*R*2 = .30; χ2(16) = 178.13, *p* < .001				

*Note.* All *R*2 are Nagelkerke. OR = odds ratio; CI = confidence interval.

Finally, we compared the average rate of turnover of prisoners across units in New Zealand prisons where a staff assault did not occur (*M* = 0.27, *SD* = 0.14), with the turnover rates of units in which a prisoner assaulted a staff member. We found that the average rate of turnover of prisoners in units where a staff assault did not occur was significantly lower than units where No Injury and Non-Serious incidents occurred: *t*(9,813) = 11.68, *p* < .001; *t*(9,502) = 19.10, *p* < .001). Units where a Serious incident occurred had lower rates of turnover of prisoners than the average rate of turnover for units in New Zealand prisons; however, they were not significantly different [*t*(9,214) = 0.27, *p* = .790]. We then examined whether rates of turnover differed depending on the security classification of the unit and found that high-security units had the highest rates of turnover compared to units of other security classifications (i.e., minimum, low medium, or maximum security units). These findings were consistent across units that housed sentenced prisoners [*F*(4) = 81.59, *p* < .001] and also in units that housed remand prisoners [*F*(3) = 175.18, *p* < .001].

## Discussion

There is limited research that examines whether the characteristics of prisoners who perpetrate staff assault differ by the level of seriousness of the incident. To address this gap, we analyzed a centralized database of physical assault incidents in to address three aims to: (a) describe the characteristic of assault incidents and their perpetrators, (b) examine their similarities and differences across three levels of assault severity, and (c) compare the characteristics of assault perpetrators with the remainder of the prison population. We also compared rates of turnover of prisoners in units where physical incidents occurred with the average rate of prisoners in any unit in a New Zealand prison.

### Perpetrator and Environmental Characteristics

We found that perpetrators of staff assault were most likely to be Māori (i.e., indigenous New Zealanders), a majority were gang affiliated, and most were classified as high security. These findings are consistent with previous research that characterizes perpetrators of staff assault as having a minority ethnic identity and being gang affiliated (Arbach-Lucioni et al., 2012; Sorensen et al., 2011; Worrall & Morris, 2012).

The most common time of day for corrections officers to be physically assaulted by a prisoner was between 8 am and 12 pm, which was somewhat consistent with previous research (Sorensen et al., 2011), and most incidents occurred in private locations (i.e., in and around cells). Activities centered around cells (i.e., unlocking or locking prisoners cells and giving prisoners food, water, or medication) increase the risk that staff will be physically assaulted; prisoners may pull staff into their cells or assault them in the doorway as staff unlock the door. However, these risky activities are integral parts of daily routines within prisons (Ellison & Gainey, 2020; McNeeley, 2021). As per the regime of the prison, staff complete these activities daily at specific times, and prisoners can predict when they will be able to interact with corrections officers. This knowledge provides an opportunity for prisoners to prepare themselves to assault a corrections officer at these key times and places, if they are motivated to do so (Light, 1991).

### Perpetrators in Directed Segregation Committed More Serious Assaults

Perpetrators of Serious incidents were more than twice as likely to be in DS prior to the incident, compared to perpetrators of no injury incidents. In our sample, around one-third of perpetrators of staff assault were housed in DS prior to perpetrating an incident. However, more than two-thirds of Serious assaults were in DS, meaning that staff assaulted in these settings are much more likely to suffer serious injuries than those assaulted elsewhere. This finding may seem surprising given that prisoners are often moved to DS because they have been judged to be at increased risk of harming others, being in DS limits the opportunities they have to commit assaults, and staff are aware and prepared for the heightened risk of harm in DS. But, on the other hand, the restricted nature of DS (i.e., having no access to programs or other structured activities, or conversation with other prisoners, and minimal time outside of their cells) means prisoners’ only social interaction may be with staff. As a result, people in DS may feel especially frustrated at their living situation, may feel like they have nothing to lose, or may simply be bored. Taken together, the history of serious violence that leads prisoners to be housed in DS and the restricted nature of DS may come together to increase the risk of violence toward staff during the periods where they are interacting.

For incidents that occurred in private locations, we found that there was a complex relationship between the prisoner turnover in the unit, the security classification of the unit, and the severity of incidents that occurred in a unit. We found that across units, assaults most commonly occurred in the high-security units and that high-security units had higher rates of turnover compared to units with other security levels (i.e., minimum, low medium, and maximum security units). Taken together, these findings may demonstrate the complexity of managing high-security prisoners in a high-security environment. Units with high turnover appear to have experienced a relatively high volume of minor assaults, perhaps because in high turnover units, greater unfamiliarity with the other prisoners and staff creates more tension or frustration. This suggestion would be consistent with previous research where high rates of turnover have been associated with unstable and tense environments in prison (Baggio et al., 2018, 2020). Although assaulting staff may lead to an increase in security classification that may cause turnover as prisoners are moved to higher security units, staff assaults are relatively infrequent compared to prisoner movements for other reasons (e.g., where beds are available, and adjustments to gang membership distributions in unit, or moving prisoners who cannot associate with each other). On the other hand, we found that a disproportionate number of Serious incidents (compared to less serious incidents) occurred in units that housed sentenced low medium prisoners, and in units with the most stable populations (i.e., units with low rates of turnover). With the information we had available for this study, we cannot be clear about potential causes for this unexpected pattern. Further investigation is needed.

### Perpetrators of Staff Assault Differed from the Total Prison Population

Consistent with previous research, we found that perpetrators of violence toward corrections officers tended to be younger than prisoners who did not perpetrate violence against corrections officers (Arbach-Lucioni et al., 2012; Cunningham et al., 2005; Lahm, 2009). The age of perpetrators is one of the most common predictors of prisoner-to-corrections officer violence (Steiner & Woolredge, 2020). Younger prisoners may have more difficulty with emotional regulation, they may be trying to prove themselves among their peers, and they may be more aggressive in general compared to older prisoners (Arbach-Lucioni et al., 2012). Younger prisoners may also be prospecting for gangs and are tasked with committing violence on behalf of the gang (New Zealand Police Association, 2021).

We found that people who were already gang affiliated were more likely to be perpetrators of assault against corrections officers than prisoners unaffiliated to gangs, which aligns with previous research that gang members typically have higher levels of misconduct in prison (Brabyn et al., 2023; Pyrooz et al., 2011; Sorensen et al., 2024; Worrall & Morris, 2012). We found that half of people who assaulted staff were affiliated with a gang, compared to one quarter of the total prison population. Gang affiliation is a risk characteristic for being involved in prison violence in general—it is commonly observed that gang members contribute to the majority of violence in prison, despite being a minority population (Pyrooz & Mitchell, 2020). Gang members as individuals typically regard themselves as responsible for maintaining the collective status of the gang, whether they are instructed to do so or not (Brennan-Tupara, 2022; Hennigan & Spanovic, 2012). They use violence to punish perceived disrespect and express anger and frustration. If a gang lets an insult go publicly unpunished, their reputation may suffer, and they may lose standing in a competitive environment. Although these dynamics are established as sources of violence toward other prisoners (Brennan-Tupara, 2022), we speculate that a gang member who feels unfairly treated by staff or perceives a staff member as disrespectful may also be more likely to use violence to communicate frustration or to punish the staff member compared to non-gang members.

It was not surprising that staff-assault perpetrators typically held higher security classifications or were housed in higher security environments prior to their assaults than prisoners in the total population, given that security status is related to the level of risk a prisoner is thought to pose toward others, and higher security environments are probably also more frustrating and stressful for prisoners and staff. We also found that a higher proportion of staff-assault perpetrators were on remand (i.e., awaiting trial or sentencing at the time of the incident) compared to the total population. In New Zealand, 44% of prisoners are on remand, and one in five is released back into the community at sentencing, because they are judged to already have spent longer in prison than the crime warranted (RNZ, 2022). Men on remand do not have a security classification; therefore, people who may eventually be classified as high risk are housed together with people who pose a low risk to others. Remand units also often have little or no access to constructive activities such as education or rehabilitation in which prisoners can engage. Men on remand may be bored and are often locked in their cells for up to 19 hr a day (Brettkelly, 2020). Anecdotally, some men have told us that they set up aggressive interactions with other prisoners to keep themselves occupied. Finally, remand units are highly stressful environments; men on remand often feel anxious about their uncertain future, legal situation, and limited contact with their families (Hatton, 2023). It seems plausible that this combination of factors all interact to produce a tense environment where violence may occurs all too frequently.

### Implications

Our findings are relevant for organizational safety; identifying characteristics of people and places that may be riskier for physically assaulting corrections staff (e.g., younger prisoners, prisoners who are gang affiliated, DS) may have implications for corrections officers when completing their daily tasks. For example, when staff complete tasks that are themselves risky in terms of assault (e.g., moving prisoners; Sorensen et al., 2011), an awareness that prisoners being younger or gang affiliated may compound this risk may enable staff to take extra precautions in these situations. These precautions may include assessing the demeanor of the prisoner before unlocking them, or ensuring that they have adequate physical measures (e.g., adequate staff) to restrain the prisoner if necessary. It is important to note that these risky activities are part of the daily regime of tasks that must be completed by staff, making it impossible to completely mitigate the risk in these necessary activities. Nevertheless, increasing staff situational awareness may be helpful for mitigating risk more generally. Recent US research indicated that correctional staff saw the potential for injury as coming from a mix of causes they could and could not control. Staff regarded complacency as a potential cause of injuries (Goulette et al., 2022); situational awareness—the antithesis of complacency—may, at the least, give staff an increased sense of control over some circumstances that may be leading to an assault.

We found that the sample of men in this study who physically assaulted corrections officers differed from the rest of the prisoner population who did not assault corrections officers and from general population of New Zealand as a whole. New Zealand has a diverse general population with 17.2% identifying as indigenous Māori and with 9% identifying as Pasifika. However, the New Zealand prison population is less diverse, which is reflected in the nature of our sample. About half of all prisoners are Māori and 16% are Pasifika, which means that New Zealand prisons are confronted with the importance of addressing the consequences of colonization and the complex needs associated with the disproportionate imprisonment of Māori (Department of Corrections, 2007). We believe that our findings may be applicable to nations with similar histories of colonization, namely Australia and Canada, with the intention of building an understanding of why Indigenous people in prison may be at higher risk of physically assaulting corrections staff.

### Limitations

The inclusion of some variables (i.e., age of perpetrator, gang affiliation) in our sample may further explain the finding that Māori people perpetrated more serious violence against staff in prison. We know that age and gang affiliation are predictive of violence perpetration both inside of and outside of prison, and we found that younger prisoners and gang-affiliated prisoners were more likely to perpetrate serious physical assault against a corrections officer. To address this issue, we conducted post-hoc analyses that revealed that Māori people in our sample were significantly younger [*F*(1) = 43.00, *p* = <.001] and significantly more likely to be gang affiliated [χ2 (1) = 129.91.21, *p* = <.001] compared to non-Māori people. It is important to acknowledge this limitation of the measurements used in our manuscript that “being *M*āori” can be viewed as proxy for other variables predictive of assault that Māori are more likely to have. This is not to say that we should ignore ethnicity as a predictor, but rather that the focus may be better placed on seeking to understand and remediate the factors—particularly those associated with the long-reaching effects of colonization—that lead to Māori being overrepresented or having higher levels of predictors both of imprisonment and of serious staff assault.

In this study, we used data from a centralized database that contained information about incidents of physical assault that occurred in New Zealand prisons. These data are limited; there was no information about the victim of the incident (aside from the fact that they were a corrections officer), and key contextual information surrounding the incident (e.g., triggers that preceded the assault, where the assault occurred, and the sequencing of events after the assault began) was also not included. Therefore, we had limited ability to compare the characteristics of those assaulted by New Zealand prisoners with previous research in other jurisdictions, information that can be helpful in developing prevention strategies that make staff and prisoners safer. It is also important to note that the incidents recorded in the centralized database are reported by corrections staff; therefore, we are also missing key information from assault perpetrators that might also inform prevention.

### Future Directions

The limitations of our research suggest the need for examining more detailed data about physical assault incidents perpetrated by prisoners toward corrections officers. We plan to examine staff incident reports of physical assaults, because these may provide more detailed data and include contextual information about the incident (i.e., precursors to the incident, the sequencing of events during the incident). Following an incident, staff must provide a written account of their perspective of the incident and their role in the incident. Although these data also have the limitation of being only from a staff perspective, analysis of the narrative content of incident reports will shed additional light on some of the content missing from this analysis. In addition to understanding how incidents occur, using incident reports we may be able to examine *how* staff respond to physical incidents. As a result, we may be able to get an understanding of what measures staff take to keep themselves and their colleagues safe in the workplace. It may also be valuable to consider other avenues of data that provide more context on prisoner to staff physical assaults. Interviewing corrections staff or even prisoners could lend insight into why assaults occur in the first place and provide a more nuanced perspective of prisoner to corrections staff assault.

## Notes

1. Fifteen of these prisons house 7,863 men; the remaining three are women’s prisons; women comprise fewer than 6% of the prison population. Typically, each prison comprises units at a variety of security levels.

2. As of June 2022, Ara Poutama employed 9,694 people, 89.8% of whom worked on the frontline in prisons. Men and women were evenly represented; more than two-thirds were European and more than one-fifth were Māori; the average age of staff was 46 years old, and the average length of service was 8 years, 9 months.

3. Turnover was calculated as follows: $\frac{\left(\alpha -\chi \right)}{\left(\beta -\chi \right)}$ , where α represents the number of unique prisoners in a unit, β represents the maximum number of months the total prisoners could be in the unit (number of prisoners × number of months in a year × number of years in the unit), and χ represents the minimum number of months prisoners could be in the unit.

4. We did not have any unit information for incidents that occurred in public areas.

5. We conducted post hoc analyses to examine the relationship between ethnicity and incident severity. A multinomial regression showed that there was a significant relationship between ethnicity and incident severity; however, this relationship was no longer significant in the presence of additional covariates (i.e., security classification, gang affiliation, custody type, and being in DS).

## References

[bibr1-08862605241287802] Ara Poutama Aotearoa. (2022). Annual Report: July 2021–30 June 2022. (Department of Corrections: Ara Poutama Aotearoa, report #E.61:).

[bibr2-08862605241287802] Ara Poutama Aotearoa. (n.d.). Prison operations manual. https://www.corrections.govt.nz/resources/policy_and_legislation/Prison-Operations-Manual

[bibr3-08862605241287802] Arbach-Lucioni K. Martinez-Garcia M. Andres-Pueyo A. (2012). Risk factors for violent behavior in prison inmates. Criminal Justice and Behaviour, 39, 1219–1239. 10.1177/0093854812445875

[bibr4-08862605241287802] Baggio S. Getaz L. Tran N. T. Peigne N. Pala K. C. Golay D. Heller P. Bodenmann P. Wolff H. (2018). Association of overcrowding and turnover with self-harm in a Swiss pre-trial prison. International Journal of Environmental Research and Public Health, 15(4), 601. 29584625 10.3390/ijerph15040601 PMC5923643

[bibr5-08862605241287802] Baggio S. Peigne N. Heller P. Getaz L. Liebrenz M. Wolff H. (2020). Do overcrowding and turnover cause violence in prison? Frontiers in Psychology, 10, 1015. 10.3389/fpsyt.2019.01015 PMC6992601 32038335

[bibr6-08862605241287802] Bakker L. O’Malley J. Riley D. (1999). Risk of reconviction. Department of Corrections.

[bibr7-08862605241287802] Boudoukha A. H. Altintas E. Rusinek S. Fantini-Hauwel C. Hautekeete M. (2012). Inmates-to-staff assaults, PTSD, and burnout: Profiles of risk and vulnerability. Journal of Interpersonal Violence, 28, 2332–2350. 10.1177/0886260512475314 23400884

[bibr8-08862605241287802] Brabyn L. Day A. Grace R. Tamatea A. (2023). Understanding prison violence in Aotearoa New Zealand using machine learning. New Zealand Geographer, 79, 234–245. 10.1111/nzg.12374

[bibr9-08862605241287802] Brennan-Tupara N. (2021). “Be feared, or live in fear”: A descriptive model of institutional gang violence (Master’s thesis, University of Waikato, Hamilton, New Zealand). Retrieved from https://researchcommons.waikato.ac.nz/entities/publication/e03cc12a-e920-4211-90a8-6e2a81ce624a

[bibr10-08862605241287802] Brettkelly S. (2020). A waiting room for danger – our remand crisis. Newsroom. Retrieved from https://newsroom.co.nz/2020/06/25/a-waiting-room-for-danger-our-remand-crisis/

[bibr11-08862605241287802] Cashmore A. W. Indig D. Hampton S. E. Hegney D. G. Jalaludin B. B. (2012). Workplace abuse among correctional health professionals in New South Wales, Australia. Australian Health Review, 36, 184–190. 22624640 10.1071/AH11043

[bibr12-08862605241287802] Christian H. (2018, June 27). Corrections officer describes working in a ‘pressure cooker’ with NZ’s worst prisoners. Stuff. https://www.stuff.co.nz/auckland/105013474/corrections-officer-describes-working-in-a-pressure-cooker-with-nzs-worst-prisoners#:~:text=%22It’s%20a%20pressure%20cooker%20right,point%20where%20it%20will%20blow.&text=%22All%20my%20fellow%20officers%20and,go%20home%20to%20our%20families.%22

[bibr13-08862605241287802] Cook C. (2020, September 1). Violence against prison guards appears to be worsening as population decreases. RNZ. https://www.rnz.co.nz/news/national/424936/violence-against-prison-guards-appears-to-be-worsening-as-population-decreases

[bibr14-08862605241287802] Crewe B. (2006). Male prisoners’ orientations toward female officers in an English prison. Punishment & Society, 8, 395–412. 10.1177/1462474506067565

[bibr15-08862605241287802] Cunningham M. D. Sorensen J. R. (2007). Predictive factors for violent misconduct in close custody. The Prison Journal, 87, 241–253. 10.1177/0032885507303752

[bibr16-08862605241287802] Cunningham M. D. Sorensen J. R. Reidy T. J. (2005). An actuarial model for assessment of prison violence risk among maximum security inmates. Assessment, 12, 40–49. 15695742 10.1177/1073191104272815

[bibr17-08862605241287802] Dennehy K. M. Nantel K. A. (2006). Improving prison safety: Breaking the code of silence. Journal of Law and Policy, 22, 175–185.

[bibr18-08862605241287802] Department of Corrections. (2007). Over-representation of Māori in the criminal justice system: An exploratory report. Policy, Strategy and Research Group: Department of Corrections.

[bibr19-08862605241287802] Duhart D. T. (2001). Violence in the workplace, 1993–99. Bureau of Justice Statistics Special Report. 12295375

[bibr20-08862605241287802] Ellison J. M. Caudill J. W. (2020). Working on local time: Testing the job-demand-control-support model of stress with jail officers. Journal of Criminal Justice, 70, 1–11. 10.1016/j.jcrimjus.2020.101717 PMC7423515 32836499

[bibr21-08862605241287802] Ellison J. M. Gainey R. (2020). An opportunity model of safety risks among jail officers. Journal of Criminal Justice, 66, 1–12. 10.1016/j.jcrimjus.2019.101632

[bibr22-08862605241287802] Fusco N. Ricciardelli R. Jamshidi L. Carleton N. Barnim N. Hilton Z. Groll D. (2012). When our work hits home: Trauma and mental disorders in correctional officers and other correctional workers. Frontiers in Psychiatry, 11, 493391. 10.3389/fpsyt.2020.493391 PMC7917131 33658946

[bibr23-08862605241287802] Giannini D. (2021). Assaults on corrections officers increase five-fold, AMC now Australia’s most expensive prison. Riotact. https://the-riotact.com/assaults-on-corrections-officers-increase-five-fold-amc-now-australias-most-expensive-prison/433802

[bibr24-08862605241287802] Gordon J. A. Proulx B. Grant P. H. (2012). Trepidation among the “keepers”: Gendered perceptions of fear and risk of victimization among corrections officers. American Journal of Criminal Justice, 38, 245–265. 10.1007/s12103-012-9167-1

[bibr25-08862605241287802] Goulette N. Denney A. S. Crow M. S. Ferdik F. V. (2022). “Anything can happen at any time”: Percevied Causes of Correctional Officer Injuries. Criminal Justice Review, 47, 17–33. https://doi.org./10.1177/0734016820952521

[bibr26-08862605241287802] Hatton E. (2023). Violent remand population blamed for increased handcuff use. Newsroom. Retrieved from https://newsroom.co.nz/2023/09/08/violent-remand-population-blamed-for-increased-handcuff-use/

[bibr27-08862605241287802] Hennigan K. Spanovic M. (2012). Gang dynamics through the lens of social identity theory. In Esbensen F. A. Maxson C. (Eds.), Youth gangs in international perspective (pp. 127–149). Springer.

[bibr28-08862605241287802] IBM Corp. (2021). IBM SPSS Statistics for Macintosh, Version 28.0. Armonk, NY: IBM Corp.

[bibr29-08862605241287802] Jaegers L. A. Ghaziri M. E. Katz I. K. Ellison J. M. Vaughn M. G. Cherniack M. G. (2022). Critical incident exposure among custody and noncustody correctional workers: Prevalence and impact of violent exposure to work-related trauma. American Journal of Industrial Medicine, 65, 500–511. 10.1002/ajim.23353 35383425

[bibr30-08862605241287802] Jaegers L. A. Matthieu M. M. Werth P. Ahmad S. O. Barnidge E. Vaughn M. G. (2020). Stressed out: Predictors of depression among jail officers and deputies. The Prison Journal, 100, 240–261.

[bibr31-08862605241287802] Johnston P. (2021). Assessing risk of re-offending: Recalibration of the Department of Corrections’ core risk assessment measure. Practice: The New Zealand Corrections Journal, 8.

[bibr32-08862605241287802] Kinman G. Clements A. J. Hart J. (2017). Working conditions, work-life conflict and well-being in U.K prison officers: The role of affective rumination and detachment. Criminal Justice and Behavior, 44, 226–239.

[bibr33-08862605241287802] Konda S. Tiesman H. Reichard A. Hartley D. (2013). U.S correctional officers killed or injured on the job. Correct Today, 75, 122–130. 26740730 PMC4699466

[bibr34-08862605241287802] Lahm K. F. (2009). Inmate Assault on Prison Staff: A multilevel examination of an overlooked form of prison violence. The Prison Journal, 89, 131–150. 10.1177/0032885509334743

[bibr35-08862605241287802] Lambert E. G. Hogan N. L. Altheimer I. (2010). An exploratory examination of the consequences of burnout in terms of life satisfaction, turnover intent, and absenteeism among private correctional staff. The Prison Journal, 90, 94–114.

[bibr36-08862605241287802] Light S. C. (1991). Assaults on prison officers: Interactional themes. Justice Quarterly, 8, 243–261.

[bibr37-08862605241287802] McNeeley S. (2021). Situational risk factors for inmate-on-staff assaults. The Prison Journal, 101, 352–373. 10.1177/00328855211010478

[bibr38-08862605241287802] Ministry of Justice. (2023). Safety in custody statistics, England and Wales: Deaths in prison custody to March 2023 assaults and self-harm to December 2022. Gov.UK https://www.gov.uk/government/statistics/safety-in-custody-quarterly-update-to-december-2022/safety-in-custody-statistics-england-and-wales-deaths-in-prison-custody-to-march-2023-assaults-and-self-harm-to-december-2022

[bibr39-08862605241287802] New Zealand Police Association. (2021). High price of gang life. https://www.policeassn.org.nz/news/high-price-of-gang-life#/

[bibr40-08862605241287802] Pyrooz D. C. Decker S. H. Fleisher M. (2011). From the street to the prison, from the prison to the street: Understanding and responding to prison gangs. Journal of Aggression, 3, 12–14.

[bibr41-08862605241287802] Pyrooz D. C. Mitchell M. M. (2020). The use of restrictive housing on gang and non-gang affiliated inmates in U.S. prisons: Findings from a national survey of correctional agencies. Justice Quarterly, 37, 590–615. 10.1080/07418825.2019.1574019

[bibr42-08862605241287802] RNZ. (2022). Court delays: 1 in 5 offenders released on time-served sentences. Retrieved from https://www.rnz.co.nz/news/national/481325/court-delays-1-in-5-offenders-released-on-time-served-sentences

[bibr43-08862605241287802] Sorensen J. R. Cihan A. Reidy T. J. (2024). Gang affiliation and prison violence: A comparison of matching analyses. The Journal of Experimental Criminology. Advance online publication. 10.1007/s11292-024-09619-8

[bibr44-08862605241287802] Sorensen J. R. Cunningham M. D. Vigen M. P. Woods S. O. (2011). Serious assaults on prison staff: A descriptive analysis. Journal of Criminal Justice, 39, 143–150.

[bibr45-08862605241287802] Spinaris C. G. Denhof M. D. Kellaway J. A. (2012). Posttraumatic stress disorder in United States corrections professionals: Prevalence and impact on health and functioning (Desert Waters Research Report). Desert Waters Correctional Outreach.

[bibr46-08862605241287802] Steiner B. Wooldredge J. (2015). Examining the sources of correctional officer legitimacy. The Journal of Criminal Law & Criminology, 105(3), 679–703.

[bibr47-08862605241287802] Steiner B. Woolredge J. (2017). Individual and environmental influences on prison officer safety. Justice Quarterly, 34, 324–349. 10.1080/07418825.2016.1164883

[bibr48-08862605241287802] Steiner B. Woolredge J. (2020). Understanding and reducing prison violence: An integrated social control-opportunity perspective. Routledge.

[bibr49-08862605241287802] Tamatea A. Day A. Cooke D. (Eds.). (2023). Preventing prison violence: An ecological perspective. Routledge.

[bibr50-08862605241287802] van Ginneken E. F. J. C. Sutherland A. Molleman T . (2017). An ecological analysis of prison overcrowding and suicide rates in England and Wales, 2000-2014. International Journal of Law and Psychiatry, 50, 76–82. https://dx.doi.org/10.1016/j.ijlp.2016.05.005 27189047 10.1016/j.ijlp.2016.05.005

[bibr51-08862605241287802] Wolff N. Blitz C. L. Shi J. Siegel J. Bachman R. (2007). Physical violence inside prisons: Rates of victimization. Criminal Justice and Behavior, 34, 588–599.

[bibr52-08862605241287802] Worrall J. L. Morris R. G. (2012). Prison gang integration and inmate violence. Journal of Criminal Justice, 40(5), 425–432. 10.1016/j.jcrimjus.2012.06.002

